# Responses of stem growth and canopy greenness of temperate conifers to dry spells

**DOI:** 10.1007/s00484-024-02682-w

**Published:** 2024-04-17

**Authors:** Jiří Mašek, Isabel Dorado-Liñán, Václav Treml

**Affiliations:** 1 Department of Physical Geography and Geoecology, Faculty of Science, Charles University, Albertov 6, 128 43 Prague, Czech Republic; 2 Dpto. de Sistemas y Recursos Naturales, Universidad Politécnica de Madrid, Madrid, Spain

**Keywords:** Tree rings, NDVI, Dry spells, Biomass allocation, Growth response, Topography

## Abstract

Dry spells strongly influence biomass production in forest ecosystems. Their effects may last several years following a drought event, prolonging growth reduction and therefore restricting carbon sequestration. Yet, our understanding of the impact of dry spells on the vitality of trees' above-ground biomass components (e.g., stems and leaves) at a landscape level remains limited. We analyzed the responses of *Pinus sylvestris* and *Picea abies* to the four most severe drought years in topographically complex sites. To represent stem growth and canopy greenness, we used chronologies of tree-ring width and time series of the Normalized Difference Vegetation Index (NDVI). We analyzed the responses of radial tree growth and NDVI to dry spells using superposed epoch analysis and further explored this relationship using mixed-effect models. Our results show a stronger and more persistent response of radial growth to dry spells and faster recovery of canopy greenness. Canopy greenness started to recover the year after the dry spell, whereas radial tree growth remained reduced for the two subsequent years and did not recover the pre-drought level until the fourth year after the event. Stem growth and canopy greenness were influenced by climatic conditions during and after drought events, while the effect of topography was marginal. The opposite responses of stem growth and canopy greenness following drought events suggest a different impact of dry spells on trees´ sink and source compartments. These results underscore the crucial importance of understanding the complexities of tree growth as a major sink of atmospheric carbon.

**Supplementary Information:**

The online version contains supplementary material available at 10.1007/s00484-024-02682-w.

## Introduction

An increasing number of extreme climatic events, mainly droughts and heat waves, significantly impact terrestrial ecosystems and their biomass production (Allen et al. 2010). Understanding trees’ responses to single or compound dry spells is crucial for estimating terrestrial carbon sequestration and carbon pools (Bonan 2008; Kannenberg et al. 2020). Carbon sequestration into forest biomass remains one of the most uncertain aspects of climate change projections simulated by Earth System Models (Friend et al. 2019), partly due to the absence of the explicit representation of growth processes within the land surface component (Zuidema et al. 2018). While plant growth in global models is computed as the difference between photosynthesis and plant respiration, direct environmental constraints on stem growth may be stronger than those on photosynthesis (Dorado-Liñán et al. 2022; Fatichi et al. 2014). As a result, the above-ground tree biomass compartments might respond to drought differently in terms of the magnitude and duration of the response. Especially at a landscape scale, where also topography matters, the coherence in responses of stems and leaves to drought spells is poorly understood.

A significant proportion of carbon is stored in aboveground compartments of trees, including the stem (35–60% of annually formed biomass) and leaves (7–16%) (Bernoulli and Körner 1999; DeLucia et al. 2000). Annual increments of stem biomass can be represented by tree-ring widths (Babst et al. 2017; Girardin et al. 2016). The greenness of leaf biomass (amount and photosynthetic activity) can be captured by vegetation indices derived from remote sensing data, such as the Normalized Difference Vegetation Index (NDVI; Vicente-Serrano et al. 2020, Song 2012) which correlates with the leaf area index (Eklundh et al. 2001) and photosynthetic activity (Zarco-Tejada et al. 2019). While time series of tree-ring width and NDVI tend to be correlated at large spatial scales (Babst et al. 2017; Vicente-Serrano et al. 2016), they differ in their climatic drivers (Seftigen et al. 2018) and may, therefore, exhibit different responses to extreme events such as dry spells (Wu et al. 2017).

The impacts of drought on tree growth are recorded not only in the year of the event (Dorado-Liñán et al. 2019; Gazol et al. 2018) but also in the following four years (legacy effects; Anderegg et al. 2015; Szejner et al. 2020). However, different tree compartments may respond differently to dry spells due to changes in biomass allocation (Zhang et al. 2015; Sevanto and Dickman 2015). The xylem, for example, depletes reserves to recover from hydraulic damage, which can suppress stem growth for several years (Trugman et al. 2018; Wu et al. 2017). In contrast, carbon investment in leaves and root formation may remain relatively unchanged during and after drought (Anderegg et al. 2015). Furthermore, different temperature thresholds are required for photosynthesis (active above 0 °C) and wood formation (active above 4–5 °C) resulting in different seasonal windows of climate sensitivity for leaf and stem biomass within a year (Fatichi et al. 2014).

In addition to the variability of responses to dry spells among biomass compartments, there might be spatial variability in the impact of drought due to microclimatic conditions connected with topographic variability (Wong et al. 2021). The curvature of the terrain, slope inclination, and orientation can affect water retention and solar irradiance which may modulate the response of trees to dry spells (Rabbel et al. 2018; Mašek et al. 2023). However, whether site topographic conditions can influence the responses of the main above-ground tree vegetative organs to dry spells at the landscape level remains unanswered.

The aim of this study is to compare the responses of wood biomass (represented by tree-ring widths) and canopy greenness (represented by the NDVI) to dry spells while accounting for the effect of topographic variability. We selected 20 plots for each of the two main coniferous tree species in Central Europe: *Picea abies* and *Pinus sylvestris* in both mountain and lowland sites with complex topography. We hypothesize that (1) the response of tree-ring widths and NDVI to dry spells will differ in terms of magnitude and duration and (2) the recovery of stem growth and canopy greenness will depend on the severity of the drought and the climatic conditions that follow, modulated by site topography.

## Methods

### Study sites and selected tree species

Coniferous forests in Central Europe primarily consist of *Picea abies* and *Pinus sylvestris* (PCAB and PISY, respectively) comprising approximately 60% of total forest coverage (Spiecker 2000). PCAB is a semi-shade tolerant, shallow-rooted tree species that typically grows in mountainous areas, forming dense closed canopies. On the other hand, PISY is a light-demanding deep-rooted species that occupies less productive sites such as sandstone and rocky slopes, where it grows in open canopies (Durrant et al. 2016).

**Fig. 1:**
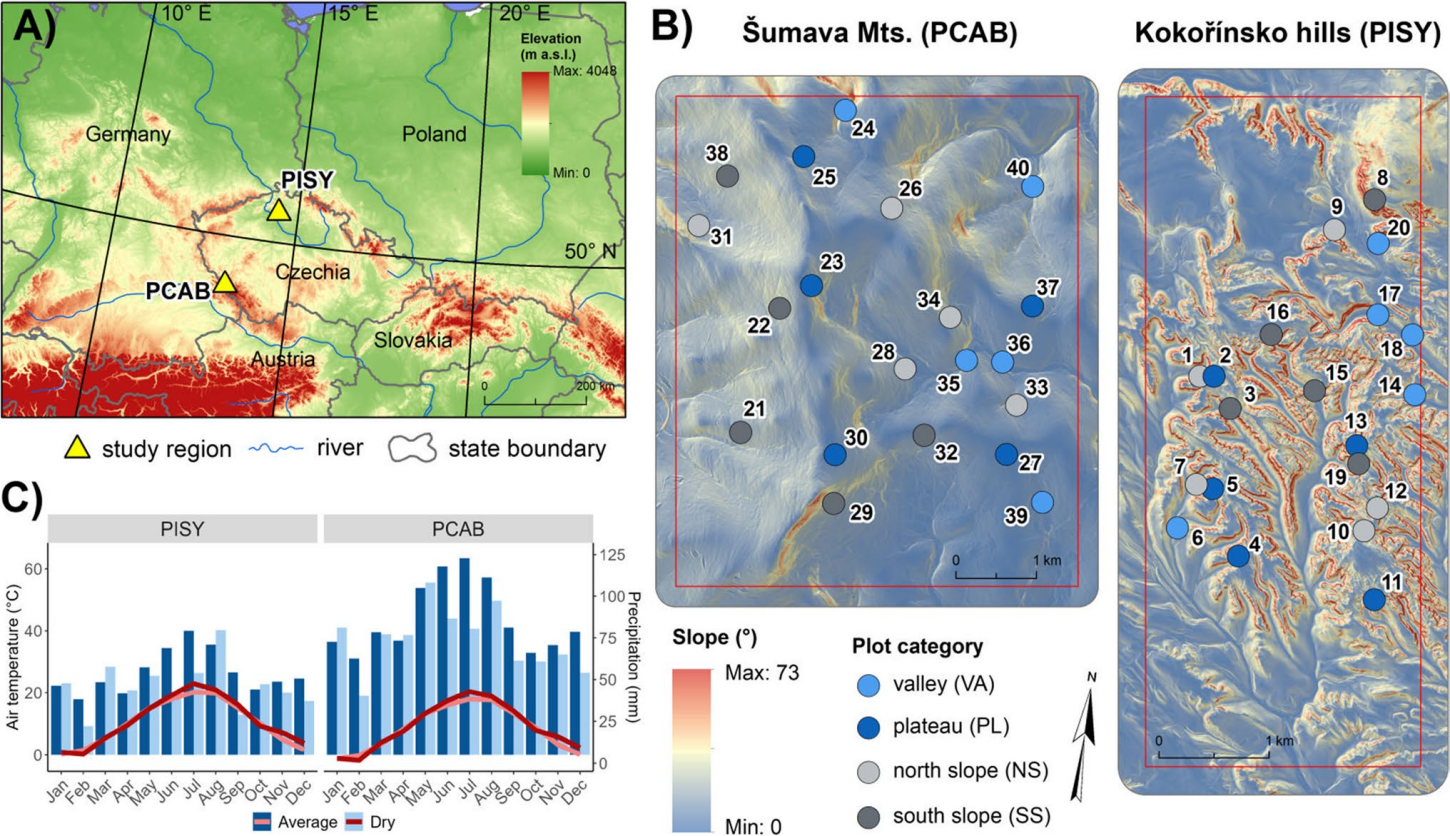
Location of two study sites dominated by *Picea abies* and *Pinus sylvestris* (PCAB and PISY respectively, panel A). Location and classification in plot categories (panel B); plot numbers correspond to those in Table S2. Composite climate diagrams for the selected drought years: 1994, 2003, 2006, and 2015 with respect to average climate conditions (1985–2017) derived from ERA5 (Hersbach et al. 2020, 2023) climatic reanalysis data (panel C)

We selected two study sites in the Czech Republic: Šumava Mts. and Kokořínsko hills which are dominated almost exclusively by PCAB and PISY, respectively (Fig. 1A). The Šumava Mts. is an old metamorphic mountain range with gentle slopes and an average elevation of 1000 m a. s. l. (Fig. S1, Table S2). The Kokořínsko hills are a sandstone platform with an elevation of approximately 400 m a. s. l. divided by deep narrow valleys where the highest peaks are formed by volcanic intrusions (Fig. 1B). The Šumava Mts. site is classified as having a wet temperate climate, with annual precipitation totals of about 1040 mm and a mean annual temperature of 5 °C. Kokořínsko hills are located in a mild temperate climate, with annual total precipitation of about 650 mm and an average annual temperature of 8 °C (period 1985–2017, Fig. 1C). In both sites, soils are generally nutrient-poor spodosols, leptosols, and cambisols (Ložek et al. 2005; Albrecht 2003).

### Sample collection and processing

We collected samples from 20 plots across each sampling site, taking into account the topographic variability and associated plot characteristics such as slope aspect, runoff, and water retention. This involved selecting five plots for each of the following plot categories: south-facing slope (plots under high solar irradiance), north-facing slope (plots under low solar irradiance), plateau (plots with low water availability), and valley bottom (plots with higher water availability; Table S1, Fig. S1) which were pre-selected based on the digital elevation model and later in the field, we narrowed down our pre-selection of particular plots also based on tree size and age. Each plot was circular, with a radius of 16 m, which is approximately equivalent to the resolution of Landsat scenes (30 m per pixel). At each plot, we sampled at least 26 mature canopy-level trees without visible damage, using a Pressler’s increment borer, a tool to extract a 5 mm cylinder of wood with minimum injury to the tree. Sampling was done in the years 2020–2021 and our dataset contains 1147 trees in total, 508 for PCAB and 639 for PISY (Table S2). The mean age of sampled trees was 113 years for both species and the mean height was 23 and 28 m for PISY and PCAB, respectively (Table S2).

We used standard dendrochronological methods to process the tree cores (Stokes and Smiley 1968). Samples were scanned with a resolution of 1200 dpi and tree-ring widths were measured using WinDENDRO (Regent Instruments 2011). Cross-dating of the tree-ring series was done in PAST 4 (Knibbe 2004) by visual verification and statistical t-test. To focus on high-frequency variability, we detrended the individual series using a 30-years-long cubic smoothing spline with a 50% frequency cut-off to remove low-to-medium frequency trends including the age trend. Tree-ring indices (TRI) were calculated as ratios between observed and fitted growth (Cook and Peters 1981). We calculated plot TRI chronologies for both species by averaging plot individual tree-ring series using Tukey's biweight robust mean (R package dplR 1.7.2; Bunn 2008).

### NDVI data

The time series of the NDVI were calculated using Landsat scenes since it provides the longest available dataset of multispectral satellite images with high resolution (30 m per pixel). We used a high-quality T1_SR collection (GEE 2023) that contains surface reflectance data and meets geometric and radiometric quality requirements from Landsat missions 5, 7, and 8. Each Landsat mission covers a different time window and uses a different sensor: Thematic Mapper, Enhanced Thematic Mapper Plus, and Operational Land Imager for Landsat 5, 7, and 8, respectively. Google Earth Engine (Gorelick et al. 2017) was used to recalculate datasets of Landsat 5 and 7 by regression to be comparable with Landsat 8 (Roy et al. 2016). The parts of the images identified as clouds and their shadows were erased to avoid distortion of spectral data in further analysis (Zhu et al. 2015).

Next, we subset all available images for the growing season period. The beginning of the growing season was defined as the first day when the mean temperature of the preceding five days exceeded 12 °C and 9 °C for PISY and PCAB plots, respectively. These temperatures are reported to trigger bud bursts for the species under study in similar elevations (Hájková 2012). Although the increment of leaf biomass is usually completed in July (Kraus et al. 2016; Fajstavr et al. 2019) the end of the growing season was set to 30th September (DOY 274) when trees in lowlands and highlands of Central Europe usually stop cell division and the tree ring is complete (Etzold et al. 2022; Tumajer et al. 2022). This approach was selected from three variants of NDVI calculations with different seasonal windows because it captures both leaf and wood phenology. Moreover, the NDVI time series did not differ considerably between different variants of calculation (Fig. S2). NDVI was calculated as follows:

1 $${\text{NDVI}}=\frac{{\text{NIR}}-{\text{Red}}}{{\text{NIR}}+{\text{Red}}}$$

Where 'Red' stands for reflectance in the red spectrum (0.64—0.67 µm) and 'NIR' indicates the reflectance in the near-infrared spectrum (0.85—0.88 µm; NASA 2022). The median of all scenes within individual years was computed and the time series of NDVI for our plots were extracted. The resulting values of the time series were calculated as the mean of pixels weighted by the proportion of the sampling plot area located inside a specific grid cell.

Time series of vegetation indices tend to be affected by numerous factors such as forest densification as trees are getting older and larger (Vicente-Serrano et al. 2004), and increasing tree and leaf size due to CO_2_ fertilization reflected also in a gradual increase in NDVI values (Donohue et al. 2013). To remove those long-term trends, we fitted a linear regression to individual NDVI series over time, and residuals from this trend line were used for calculating a mean NDVI series per plot.

NDVI tends to saturate during the growing season, particularly in evergreen conifer forests. However, in our case, the values of NDVI are slowly increasing each year and never reach full saturation (Fig. S3). We are aware, that although we tried to carefully select monospecific undisturbed plots, our NDVI data might be affected by distortion of spectral data due to admixed vegetation, and in the case of open PISY canopies, there might be also a signal of understory and soil. However, the adjacent pixels of Landsat scenes returned almost identical time series, so the signal of understory vegetation is probably of limited significance.

### Climate data

We used ERA5 atmospheric reanalysis data (Hersbach et al. 2020, 2023) with a spatial resolution of 0.25° per pixel. ERA5 data describes general macroclimatic settings for both sites and the deviations due to orographic differences are represented by categorical variables characterising distinct topoclimatic variations (valleys, slopes, summits; Geiger et al. 2009). Monthly data on air temperature (T in °C), precipitation (P in mm), downward surface solar radiation (SR in J/m^2^), and soil moisture in 10 cm (SM in kg/ m^2^) were obtained from Climate Earth Explorer. Based on T and P we also calculated the Standardised Precipitation-Evapotranspiration Index (SPEI, Vicente-Serrano et al. 2010) using the Thornthwaite method (Thornthwaite 1948). We considered different durations of the preceding period spanning from 1 to 12 months for the calculation of SPEI (R package SPEI 1.7, Beguería and Vicente-Serrano 2017). Since there were negligible differences in correlations between TRI or NDVI with different SPEI versions, we selected SPEI with four preceding months since it represents a balance for different rooting strategies between species and depth of water uptake. While deeply rooting species (PISY) can use water retained in the soil most of the year, shallow rooting species (PCAB) reach subsurface layers of soil with a fast turnover of infiltrating precipitation (Sprenger et al. 2019).

We computed the mean climatic series for summer (June, July, August; JJA) and the growing season (May through September; GS). Since the results for JJA and GS showed little differences, only JJA results are shown in the main body of the text.

### Statistical analysis

We selected the time window of 1985–2017 as the common period for analyses, which was covered by both TRI and NDVI time series. All analyses were performed using R software (R 4.2.0; R Core Team 2022).

To identify the main climate drivers, we calculated Pearson’s correlations between TRI, NDVI chronologies, and climate variables in the current and previous year. Since SR and SM showed the highest correlations with TRI and NDVI, the most severe non-consecutive drought years were selected based on the lowest (SM) and the highest (SR) values. Three of the four resulting drought years overlapped for both SM and SR. For simplicity, we decided to use those derived from SR because they were identical for both tree species. Non-consecutive drought years were selected to avoid the potential cumulative effect of successive drought years on tree growth (Anderegg et al. 2020; Gessler et al. 2020).

To explore the response of stem (TRI) and canopy greenness (NDVI) to dry spells, we employed superposed epoch analysis (SEA; Chree 1913) which calculates the significance of the deviation from the mean of a given year and several lagged years. We used the 'sea' function in R package dplR (1.7.2; Bunn 2008) and considered a four-year lag before and after the dry spell (Anderegg et al. 2015; Wu et al. 2017).

Additionally, we developed linear mixed-effect models (R package lme4 1.1–30; Bates et al. 2015) with TRI and scaled NDVI in a given drought year and the four following years as the dependent variables. Explanatory variables were the severity of solar radiation in the drought year (anomaly from average SR; SEV0), the solar radiation severity in each of the four years following the dry spell (anomaly from average SR; SEV), while the plot category was treated as a random effect. For each variable in all models, we calculated the variance inflation factor (VIF; R package ‘car’; 3.1–1; Fox and Weisberg 2019). We created three variants of models: a full model with all predictors and two models always with a single predictor omitted (successively: SEV0, SEV). We assessed the performance of each model using the Akaike information criterion (AIC), marginal and conditional R^2^ (R package MuMIn 1.47.1; Barton 2022), pseudo-R^2^, t values, and p-values of all predictors. The significance of the random effect (plot category) was calculated by the function 'ranova' from R package lmerTest (3.1–3; Kuznetsova et al. 2017). Finally, we checked the normal distribution of residuals using qqplots.

## Results

### Climatic signal

Both species and biomass compartments showed negative correlations with seasonal temperatures and surface solar radiation while precipitation, soil moisture, and SPEI had a positive influence on productivity (Fig S4). PCAB TRI primarily reflected the influence of the previous year's climate, whereas NDVI appeared to be minimally affected by climate. There was no difference between plot categories. PISY NDVI showed the strongest climate signal, displaying significant correlations with all climatic variables, especially with SM and SR, with no differences between plot categories. PISY TRI exhibited the most significant correlations in plots located on plateaus, whereas correlations in other plot categories were less or non-significant (Fig. S4).

### Responses to dry spells

**Fig.2:**
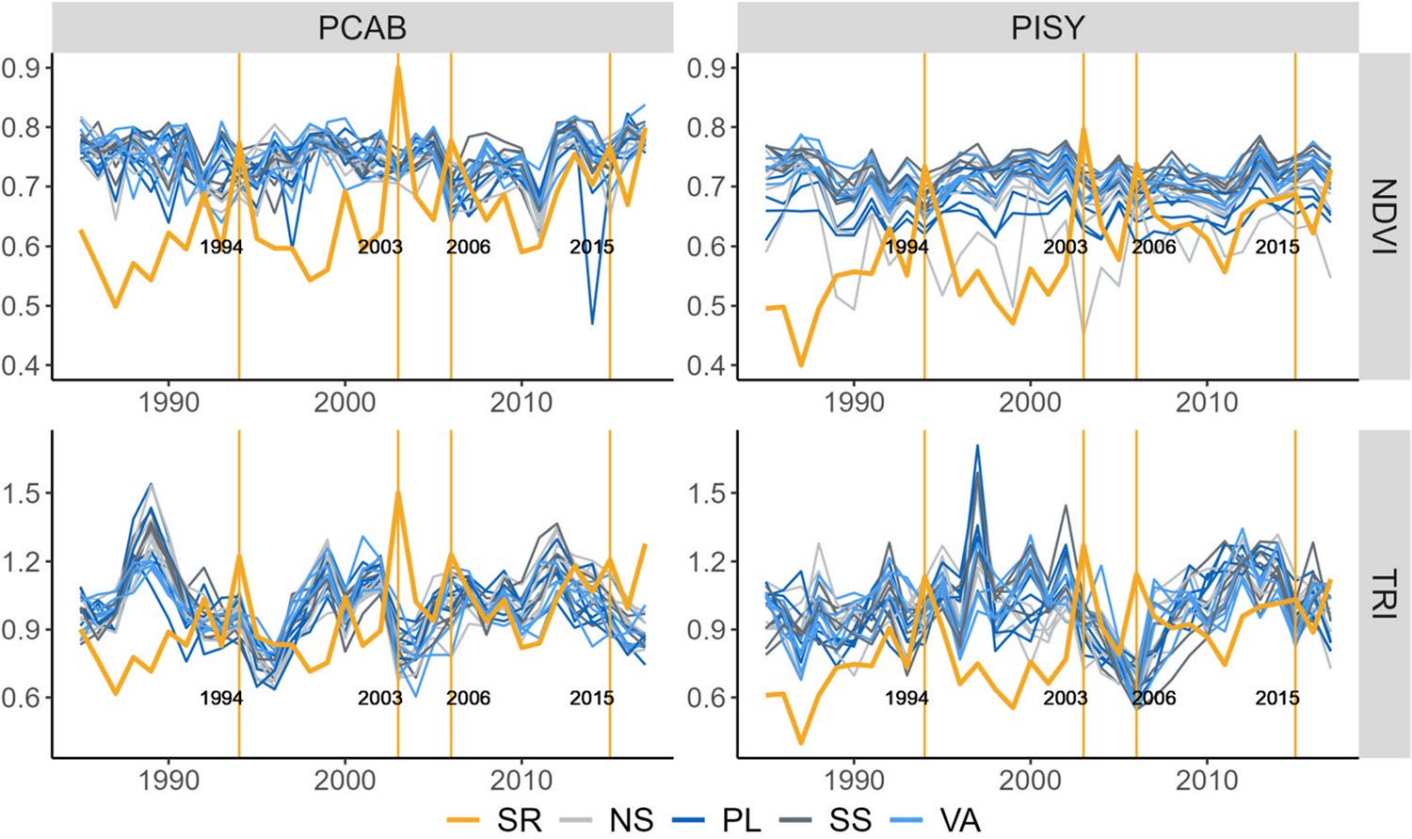
Time series of normalized difference vegetation index (NDVI; upper panel) and tree-ring indices (TRI; lower panel) for each plot and tree species: *Picea abies* (PCAB; left panel), and *Pinus sylvestris* (PISY; right panel). Colors indicate the plot categories (SS-South slops, NS-North slope, PL-Plateau, VA-Valley). The orange line corresponds to the surface solar radiation time series (SR) and vertical lines highlight the selected drought years

**Fig. 3:**
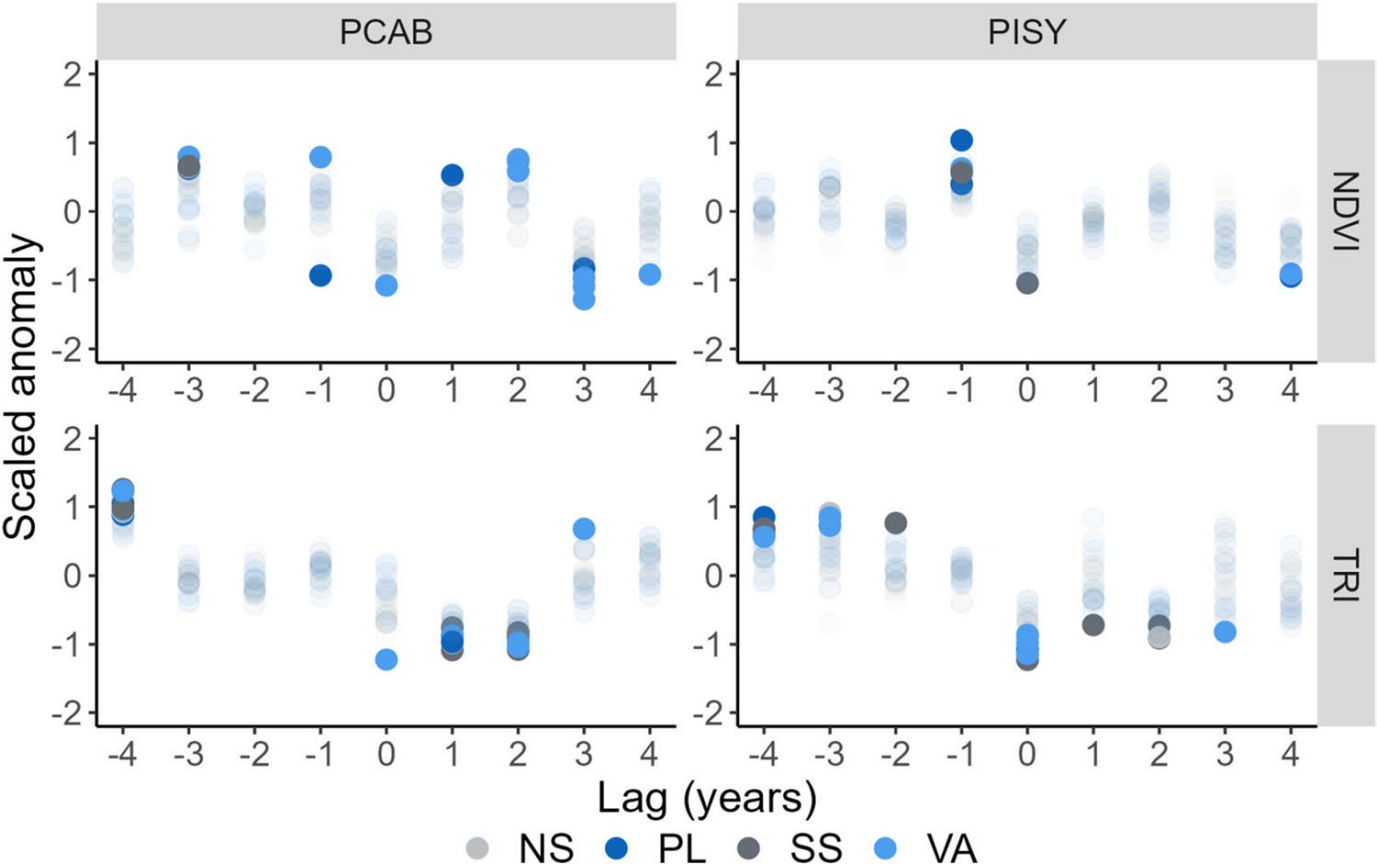
Responses of normalized difference vegetation index (NDVI; upper panel) and tree-ring indices (TRI; lower panel) to drought years for *Picea abies* (PCAB; left panel) and *Pinus sylvestris* (PISY; right panel) as indicated by superposed epoch analysis. Solid dots denote statistically significant change (p < 0.05) and color indicates the plot category (SS-South slope, NS-North slope, PL-Plateau, VA-Valley)

In both sites, the identified driest years were 1994, 2003, 2006, and 2015 (Fig. 2). The response of PCAB TRI to these dry spells exhibited large variability among individual plots (Fig. 3). However, a synchronized and significant decrease in tree growth lasting the following two years affected almost all plots irrespective of the plot category. The recovery of tree growth is visible from the third post-drought year onwards when trees reached pre-drought levels of growth (Fig. 3). PISY TRI significantly dropped in the year of the drought event at most of the plots. The growth reduction lasted for the next two years, but only significantly for the slope plots (i.e., north and south slope plots; Fig. 3). At some PISY plots, trees did not recover to the pre-drought radial growth level four years after the dry spell.

For both tree species, we observed a significant reduction in NDVI during drought events. In PCAB, this reduction was followed by a significant increase in NDVI two years after the dry spell, achieving even higher levels of greenness than before the drought event (Fig. 3). However, three years after the dry spell, the NDVI dropped, and this change was significant for most PCAB plots. Conversely, PISY did not recover pre-drought NDVI levels four years after the drought event. Overall, there were no differences in the response between plot categories, irrespective of species or TRI and NDVI.

### Factors influencing the responses of TRI and NDVI

The models for PISY TRI explained approximately 24% of the variability in the data, with the climate severity in the year of the event (Severity0) and the years following the drought event (Severity) emerging as significant predictors (Table 1). In turn, the models for PCAB TRI captured approximately 12% of data variability with no significant influence of any predictor. Models for PISY NDVI accounted for 28% of the variability with both climate severities (Severity0 and Severity) demonstrating significant effects. In the case of PCAB NDVI, approximately 21% of the variability was explained, with both climate severities being statistically significant. In all cases, the explained variability of the models decreased when Severity0 was omitted. Models without the Severity variable also exhibited lower explained variance compared to full models (except the model for PCAB TRI) but the decrease was substantially smaller compared to models without Severity0 (Table 1). All predictors in all models displayed VIF below 2 and a distribution of residuals close to normal.

**Table 1:** Results of linear mixed-effect models explaining normalized difference vegetation index (NDVI) and tree-ring indices (TRI) for *Pinus sylvestris* (PISY) and *Picea abies* (PCAB). The columns indicate the Akaike information criterion (AIC), R^2^ marginal, R^2^ conditional, pseudo-R^2^, and t-values of predictors (Severity0 and Severity). Bold numbers indicate significant p values (p < 0.05)

SPECIES	VAR	Model	AIC	R^2^ marginal	R^2^ conditional	pseudo R^2^	Severity0	Severity	Plot category
PISY	**TRI**	**Full model**	-251.647	0.289	0.311	0.315	**-7.253**	**-3.261**	0.998
		**without SEV0**	-215.165	0.111	0.114	0.115	NA	**-5.975**	0.993
		**without SEV**	-258.734	0.266	0.287	0.291	**-7.377**	NA	0.973
	**NDVI**	**Full model**	720.300	0.435	0.466	0.469	**-9.338**	**-10.365**	0.883
		**without SEV0**	841.901	0.142	0.142	0.143	NA	**-7.722**	1.000
		**without SEV**	827.109	0.218	0.241	0.243	**-9.313**	NA	0.936
PCAB	**TRI**	**Full model**	-469.296	0.159	0.207	0.209	1.535	-0.894	0.910
		**without SEV0**	-447.903	0.000	0.000	0.000	NA	-0.004	1.000
		**without SEV**	-487.156	0.157	0.199	0.201	1.505	NA	0.699
	**NDVI**	**Full model**	860.422	0.356	0.356	0.358	**-9.563**	**-8.411**	1.000
		**without SEV0**	959.970	0.054	0.054	0.054	NA	**-4.520**	1.000
		**without SEV**	908.825	0.229	0.229	0.230	**-9.232**	NA	1.000

## Discussion

We combined the analysis of TRI (a proxy for stem growth dynamics) and NDVI (a proxy for canopy greenness) for two conifer species (*Pinus sylvestris* and *Picea abies*) in Central Europe growing under different microenvironments to comprehensively characterize their response to drought. Our results suggest that following dry spells, conifers undergo a systematic shift in physiological activity between stem and leaf. The growth of trees during the drought year and the three following years is influenced by climatic conditions while the effect of topography is marginal.

### Differential response of TRI and NDVI to dry spells

Our sampling sites are landscapes characterized by complex topography, associated with high spatial variability in solar irradiance and water availability. The PISY site, located in Kokořínsko hills, experiences warm and dry conditions leading to pronounced drought effects on both stem growth and leaf greenness in trees. In contrast, the PCAB site in the Šumava Mountains encounters climate conditions that closely align with the PCAB climatic optimum, resulting in a weaker influence of climate on both analyzed proxies, TRI and NDVI,.

Consistent with previous studies, the impact of a dry spell in both TRI and NDVI persisted for three years following the event in both tree species (Wu et al. 2017; Szejner et al. 2020; Janecka et al. 2022). The impact of the drought event on PISY TRI was more pronounced than on PCAB TRI, which can be explained by the higher moisture limitation of the PISY site compared to the PCAB site (Fig. S4). The response of PCAB TRI in the years after the dry spell was similar to that of PISY, though the reduction was more significant and persistent for most of the PCAB plots (Marchand et al. 2021; Zlobin 2022). This might be due to the different rooting strategies of both species (Durrant et al. 2016). Shallow-rooting species such as PCAB are assumed to be more sensitive to dry spells than deep-rooting PISY, which buffers against drought effects by accessing groundwater in deeper soil layers (Kannenberg et al. 2020; Mackay et al. 2020). Differences may also be related to the higher isohydricity of PCAB compared to relatively more anisohydric PISY (Martínez-Vilalta et al. 2014) leading to stronger regulation of stomatal conductance in PCAB and thus production of less assimilates and storage sugars compared to PISY. Furthermore, PCAB TRI growth is negatively influenced by the temperature of the preceding summer and positively impacted during the winter (Fig. S4; Mašek et al. 2023). Winter conditions may interact with the preceding summer and late autumn weather, leading to the observed strong negative growth responses over two consecutive years (Harvey et al. 2020).

After dry spells, stem growth (i.e., TRI) was reduced and this reduction persisted for two years, whereas NDVI, which is a proxy integrating photosynthetic activity and leaf biomass, increased for both species during the same period (Fig. 3). This suggests a stem-leaf biomass trade-off. According to our results, trees changed their allocation strategy in the years following dry spells, probably in order to invest more carbon into the leaf biomass to enhance photosynthesis (replenish sugar pools) and restore canopy damage (Kannenberg et al. 2019a; Anderegg et al. 2013). Such an NDVI enhancement was more significant for PCAB which might be connected to a stronger reduction of TRI observed for this species. Increases in forest ecosystems’ NDVI in years following a dry spell were also observed and reported by other studies (Rita et al. 2020; Dong et al. 2022; Gazol et al. 2022), evidencing that responses of stem growth and leaf biomass greenness to dry spells are uncoupled (Gazol et al. 2020; Moreno-Fernández et al. 2022).

The larger reduction of stem growth compared to canopy greenness during drought events (Fig. S3) has been previously reported by other studies using canopy vigor proxies derived from remotely-sensed vegetation indices or eddy covariance data (Kannenberg et al. 2020, 2019a; Moreno-Fernández et al. 2022). All these findings reveal that stem growth during a dry spell is more limited by climate conditions than canopy greenness (Cabon et al. 2022; Dow et al. 2022). Wood formation stops at higher plant water potential than photosynthesis (vegetation greenness) since radial tree growth is limited by a low turgor in cambial cells (Cabon et al. 2020). Low water potential also reduces the ability to transport assimilated carbon (Fatichi et al. 2014), leading to the accumulation of non-structural carbohydrates in trees that might be invested in other tree organs than stems such as roots (Teets et al. 2022; Lapenis et al. 2013), respired or stored in carbon pools. In the end, all these factors might contribute to an overall more pronounced response of stem compared to canopy greenness in the drought year.

In the two years following the dry spell, the canopy greenness is restored probably at the expense of stem growth (Anderegg et al. 2013). Only in the third year after the dry spell, does the stem growth return to a normal level (Martínez-Sancho et al. 2022; Anderson-Teixeira and Kannenberg 2022) assuring sufficient conductive capacity for leaves (Kröber et al. 2014), with some indications that this is accompanied by a simultaneous decrease of NDVI values. We can speculate that stem growth is enhanced at the expense of leaf biomass in the third year after a dry spell, however, our data doesn’t provide direct evidence. In the fourth year, both biomass compartments recovered back to the pre-drought values of TRI and NDVI (Klesse et al. 2022; Szejner et al. 2020; Leifsson et al. 2023). Detected differences between tree rings and NDVI in this study imply partial decoupling of stem and leaf biomass responses and probably also varying carbon allocation strategies after dry spells.

All selected dry spells (1994, 2003, 2006, 2015) are well-known to have affected plant growth in Central Europe (Spinoni et al. 2015; Moravec et al. 2021), but they differ in their meteorological characteristics. The variability in drought severity, duration, and timing leads to significant differences in the response of both stem growth and canopy greenness to individual dry years (Gao et al. 2018; Huang et al. 2018; Kannenberg et al. 2019b; Wu et al. 2022) and hence, in trees’ resilience, resistance and recovery (Text S1, Table S3, Fig. S5).

### Factors shaping the responses to drought

We hypothesized that drought severity and topography modulate the response of trees to dry spells. Our results suggest that drought severity is a very important factor, while the topography has a limited effect in our dataset.

The severity of the dry spell and the climatic severity during the following years were highly significant predictors of stem growth and canopy greenness for both studied species, corroborating the results of other studies for conifers and broadleaf species from the temperate biome (Brun et al. 2020; Song et al. 2022; Castellaneta et al. 2022; Meng et al. 2015). Differences in the response to dry spells might be partly caused by different rooting strategies of species under study, since root properties such as root length, fine root diameter, and root density may significantly influence drought tolerance (Chen et al. 2022). Deep-rooting has been observed as a mitigation strategy in response to drought (Mohammadi Alagoz et al. 2022; Chitra-Tarak et al. 2021).

Overall, the influence of topography was rather marginal (Schmied et al. 2023, Table 1). Likely, drought severity outperforms the potential effect of topography. Alternatively, the impact of dry spells was so severe that even the conditions in valleys (water accumulation) were not favourable enough to provide a sufficient amount of soil moisture. This means that during mild droughts, the topography may play a role as seen in the radial growth of trees growing in topographically distinct conditions (Strum et al. 2022; Rabbel et al. 2018). We further observed highly variable response of PISY TRI in SEA (Fig. 3) which might be a result of complex topographic conditions in Kokořínsko hills with relatively greater between-plot differences (Fig. 1). However, if the response to drought was assessed using resilience metrics, there was no difference between the plot categories for any species or biomass compartment (Fig. S5) confirming the marginal effect of topography during extreme droughts.

At each site we used ERA5 climate data from one grid cell. Using the same climate data for all plots may mask some topography-related differences between them. However, since our analyses do not depend on absolute values of climatic variables, the potential impact of using the same data for all plots is low. We assume that the values of monthly temperature and surface solar radiation used in our analyses reveal a systematic shift between valleys, slopes, and summits (Geiger et al. 2009; Daly et al. 2010; Treml and Banaš 2008), which is represented by categorical variables characterizing the topographic position of each plot.

## Conclusions

Forest responses to drought are complex with potential differences among tree species and tree biomass compartments, and across topographically complex landscapes. We focused on the responses of stem growth (represented by tree-ring widths) and canopy greenness (represented by NDVI) to a drought of two important coniferous tree species (*Pinus sylvestris* and *Picea abies*) in Central Europe. Our findings reveal a decoupled response of stem growth and canopy greenness in the period following dry spells suggesting the importance of changing carbon allocation strategies during and after drought. Moreover, the magnitude of the response to drought spells was also species-specific: stem growth reduction and NDVI increase of deep-rooting *Pinus sylvestris* were not as conspicuous as those of the shallow-rooting *Picea abies*. Furthermore, drought severity appears to outweigh any potential variations in response linked to topography, as the influence of landscape features was marginal. We demonstrated the decisive role of drought severity in the response of above-ground tree compartments, which, in turn, exhibit systematic differences in the recovery period. The impact of dry spells on above-ground tree biomass compartments in temperate conifer forests is primarily driven by the drought severity followed by biomass allocation strategy, tree species, and landscape topography. Understanding the carbon allocation strategies triggered by dry spells is crucial for forecasting changes in forest ecosystems and improving our knowledge of forest responses to extreme droughts.

### Supplementary Information

Below is the link to the electronic supplementary material.

Supplementary file1 (DOCX 1665 KB)

## Data Availability

Tree-ring width data have been uploaded to the International Tree-Ring Data Bank (ITRDB). Codes for NDVI time-series calculation in Google Earth Engine are provided here: PCAB: https://code.earthengine.google.com/ddb1fc420d3be0f01719effb1facfa2b PISY: https://code.earthengine.google.com/8a54269c4279d7795aeec9cc5873d109 All used data (climate, ring width, and NDVI) are available for download on GitHub (https://github.com/JirkaSkaut/Responses-to-dry-spells) including the R script for all calculations and figures.
