# Supplementary material for: Responses of stem growth and canopy greenness of temperate conifers to dry spells
Source: Int J Biometeorol. 2024 Apr 17;68(8):1533–44. doi: 10.1007/s00484-024-02682-w (PMC11281975; doi:10.1007/s00484-024-02682-w)

**Responses of stem growth and canopy greenness of temperate conifers to dry spells**

Jiří Mašek^a*^, Isabel Dorado-Liñán^b^, Václav Treml^a^

^a^ Department of Physical Geography and Geoecology, Faculty of Science, Charles University, Albertov 6, 128 43 Prague, Czech Republic

^b^ Dpto. de Sistemas y Recursos Naturales, Universidad Politécnica de Madrid, Madrid, Spain.

^*^ Corresponding author: jiri.masek@natur.cuni.cz (Jiří Mašek)

**Supplementary material**

**Fig S1:** Topographic characteristics (ALT-Altitude, HLI-Heath load index, NOR_DEV-Deviation from north, SLOPE-Slope, TWI-Topographic wetness index) of plot categories (SS-South slope, NS-North slope, PL-Plateau, VA-Valley) for Pinus sylvestris (PISY) and Picea abies (PCAB). For specifications of HLI, NOR_DEV, and TWI please see Table S1.

**
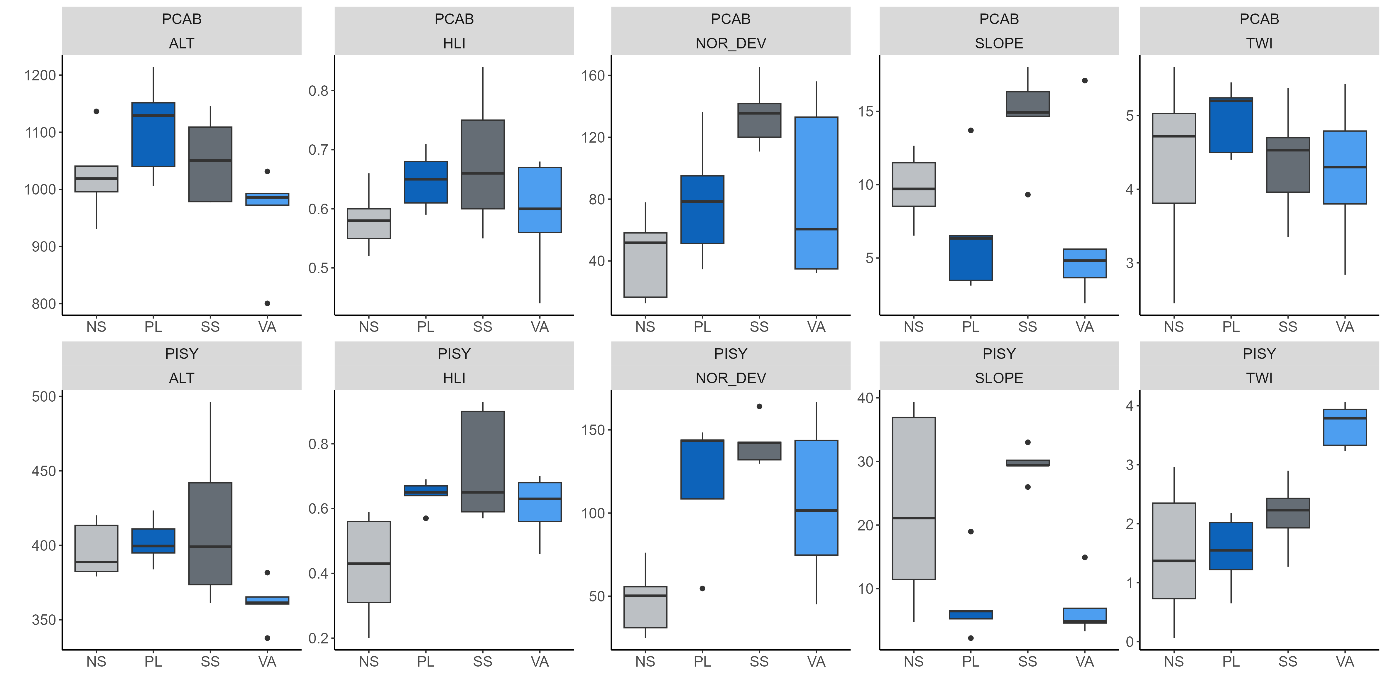
**

**Table S1:** Topographic characteristics (Heath load index-HLI; North deviation-NOR_DEV; Topographic wetness index-TWI) specifications and equations. Calculations were performed on LIDAR-based surface topography models with a regular grid of 5 x 5 m (DMR 4G; ČÚZK 2013) using ArcMap 10.7.1 (ESRI 2020).

| **Variable** | **Description** | **Equation** | **Reference** |
| --- | --- | --- | --- |
| **Heath load index (HLI)** | HLI represents the capacity of a given place for heating (high values) or cooling (low values) due to favorable slope, orientation, and latitude | HLI=EXP[-1.467+1582*(cos(L)*cos(S) )-1.5*(cos(A)* sin(S)* sin (L))-0.262*(sin(L)* sin(S))+0.607*(sin(A) * sin(S))]  L=latitude (rad)  S=slope (rad)  A=180-∣ASP-180∣ (rad)  ASP = aspect of slope (deg) | McCune and Keon (2002) |
| **North deviation (NOR_DEV)** | NOR_DEV represents the insolation of a given place due to orientation to the north (low values) or to the south (high values) | NOR_DEV = ((ASP<180)*ASP)+∣(ASP>180)*(APS-360)∣  ASP = aspect of slope (deg) |  |
| **Topographic wetness index (TWI)** | TWI represents a topographically induced predisposition of a given place for moisture accumulation (high values) | TWI = ln(α/tan(β))  α = flow accumulation  β = surface slope (rad) | Beven and Kirkby (1979) |

**Table S2:** General plot characteristics of Pinus sylvestris (PISY) and Picea abies (PCAB). Study plots, ordered according to species and plot category.

| Site no. | Species | Site category | Coordinates: Lat; Long (°) | Elevation (m a. s. l.) | No. of trees | Mean  age  ± SD (year) | Mean DBH ± SD (cm) | Mean height ± SD (m) |
| --- | --- | --- | --- | --- | --- | --- | --- | --- |
| 1 | PISY | North slope | 50.583; 14.432 | 388,79 | 26 | 66 ± 41 | 33 ± 7 | 23 ± 3 |
| 7 | PISY | North slope | 50.574; 14.433 | 379,11 | 26 | 118 ± 20 | 35 ± 7 | 18 ± 3 |
| 9 | PISY | North slope | 50.596; 14.447 | 382,54 | 26 | 78 ± 34 | 37 ± 6 | 26 ± 3 |
| 10 | PISY | North slope | 50.572; 14.455 | 420,16 | 65 | 122 ± 88 | 34 ± 6 | 11 ± 3 |
| 12 | PISY | North slope | 50.574; 14.457 | 413,36 | 26 | 149 ± 11 | 36 ± 7 | 27 ± 4 |
| 2 | PISY | Plateau | 50.583; 14.434 | 411,01 | 27 | 128 ± 42 | 38 ± 4 | 18 ± 3 |
| 4 | PISY | Plateau | 50.569; 14.440 | 383,86 | 26 | 128 ± 36 | 31 ± 5 | 17 ± 2 |
| 5 | PISY | Plateau | 50.574; 14.436 | 394,91 | 26 | 148 ± 19 | 25 ± 5 | 17 ± 3 |
| 11 | PISY | Plateau | 50.567; 14.458 | 399,54 | 59 | 158 ± 80 | 35 ± 8 | 9 ± 2 |
| 13 | PISY | Plateau | 50.579; 14.453 | 423,42 | 25 | 138 ± 9 | 23 ± 10 | 22 ± 2 |
| 3 | PISY | South slope | 50.580; 14.436 | 373,56 | 26 | 78 ± 31 | 22 ± 9 | 28 ± 5 |
| 8 | PISY | South slope | 50.599; 14.451 | 496,45 | 64 | 125 ± 11 | 37 ± 7 | 15 ± 3 |
| 15 | PISY | South slope | 50.583; 14.447 | 361,25 | 26 | 94 ± 45 | 34 ± 5 | 29 ± 5 |
| 16 | PISY | South slope | 50.587; 14.440 | 442,00 | 26 | 143 ± 16 | 46 ± 8 | 26 ± 4 |
| 19 | PISY | South slope | 50.577; 14.454 | 399,07 | 26 | 144 ± 12 | 39 ± 7 | 23 ± 3 |
| 6 | PISY | Valley | 50.570; 14.432 | 337,70 | 26 | 73 ± 17 | 42 ± 6 | 30 ± 3 |
| 14 | PISY | Valley | 50.584; 14.460 | 360,62 | 35 | 119 ± 8 | 42 ± 5 | 36 ± 6 |
| 17 | PISY | Valley | 50.590; 14.454 | 365,34 | 26 | 110 ± 17 | 43 ± 4 | 30 ± 3 |
| 18 | PISY | Valley | 50.588; 14.458 | 361,48 | 26 | 61 ± 5 | 36 ± 4 | 31 ± 5 |
| 20 | PISY | Valley | 50.595; 14.452 | 381,66 | 26 | 94 ± 21 | 42 ± 6 | 26 ± 3 |
| 26 | PCAB | North slope | 49.211; 13.260 | 1136,41 | 26 | 160 ± 9 | 47 ± 8 | 26 ± 3 |
| 28 | PCAB | North slope | 49.193; 13.266 | 1018,92 | 26 | 101 ± 3 | 54 ± 8 | 29 ± 4 |
| 31 | PCAB | North slope | 49.206; 13.227 | 930,87 | 23 | 99 ± 17 | 44 ± 10 | 35 ± 3 |
| 33 | PCAB | North slope | 49.191; 13.286 | 995,72 | 25 | 123 ± 30 | 47 ± 7 | 30 ± 4 |
| 34 | PCAB | North slope | 49.200; 13.272 | 1040,71 | 26 | 94 ± 19 | 38 ± 7 | 32 ± 3 |
| 23 | PCAB | Plateau | 49.201; 13.248 | 1214,19 | 25 | 93 ± 21 | 40 ± 8 | 19 ± 2 |
| 25 | PCAB | Plateau | 49.215; 13.243 | 1129,26 | 26 | 101 ± 13 | 47 ± 8 | 22 ± 2 |
| 27 | PCAB | Plateau | 49.186; 13.285 | 1039,89 | 26 | 117 ± 10 | 40 ± 7 | 30 ± 3 |
| 30 | PCAB | Plateau | 49.183; 13.256 | 1151,50 | 25 | 113 ± 12 | 47 ± 9 | 22 ± 3 |
| 37 | PCAB | Plateau | 49.203; 13.286 | 1005,97 | 26 | 87 ± 5 | 35 ± 7 | 30 ± 3 |
| 21 | PCAB | South slope | 49.184; 13.240 | 977,47 | 26 | 111 ± 10 | 52 ± 10 | 28 ± 2 |
| 22 | PCAB | South slope | 49.198; 13.243 | 1145,46 | 26 | 123 ± 16 | 44 ± 9 | 21 ± 2 |
| 29 | PCAB | South slope | 49.178; 13.257 | 1108,93 | 26 | 118 ± 13 | 45 ± 9 | 32 ± 4 |
| 32 | PCAB | South slope | 49.187; 13.271 | 1050,51 | 24 | 108 ± 10 | 51 ± 10 | 29 ± 4 |
| 38 | PCAB | South slope | 49.211; 13.231 | 978,54 | 25 | 132 ± 17 | 36 ± 9 | 33 ± 3 |
| 24 | PCAB | Valley | 49.221; 13.249 | 1031,37 | 25 | 179 ± 27 | 42 ± 11 | 30 ± 2 |
| 35 | PCAB | Valley | 49.195; 13.276 | 985,95 | 25 | 150 ± 30 | 40 ± 7 | 26 ± 6 |
| 36 | PCAB | Valley | 49.196; 13.282 | 972,35 | 26 | 103 ± 29 | 55 ± 12 | 28 ± 3 |
| 39 | PCAB | Valley | 49.181; 13.292 | 992,90 | 25 | 100 ± 12 | 38 ± 14 | 26 ± 5 |
| 40 | PCAB | Valley | 49.216; 13.283 | 800,47 | 26 | 66 ± 6 | 44 ± 8 | 32 ± 4 |

**Figure S2:** Correlations between normalized difference vegetation index (NDVI) for the individual plots of Pinus sylvestris (PISY) and Picea abies (PCAB). Colors denote pairs of settings of the growing season: May–September (MS), Jun–August (JA), and the variable start of the growing season for each year based on daily climate data to September (DOY 274, series presented in this paper).


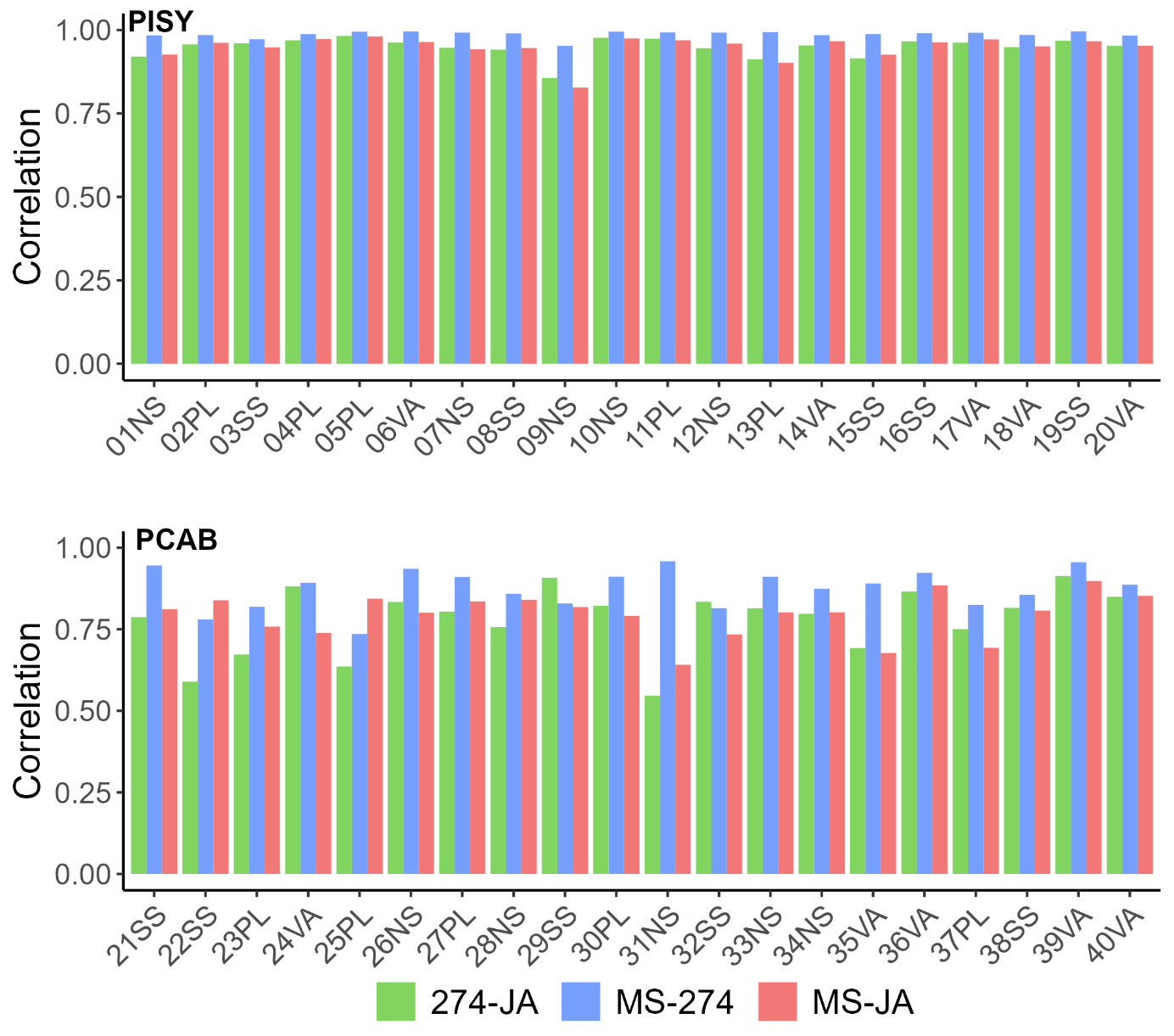


**Figure S3:** NDVI values of individual Landsat scenes during the vegetation period (from the day of bud burst to 30th September) for random points within studied sites for Pinus sylvestris (PISY) and Picea abies (PCAB). Colors denote individual years.

**
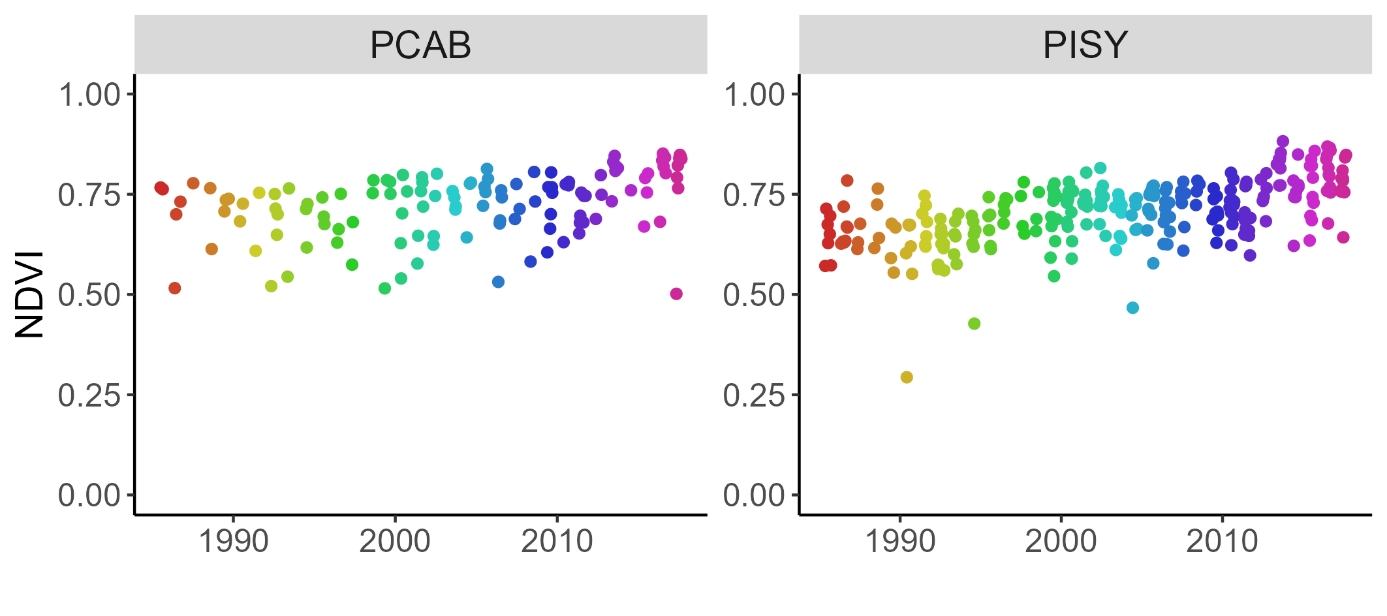
**

**Figure S4:** Mean correlation (bars) and standard deviations (error bars) of normalized difference vegetation index (NDVI; upper panel) and tree-ring width (TRI; lower panel) for Picea abies (PCAB; left panel) and Pinus sylvestris (PISY; right panel) with climatic variables (colors; P-Precipitation, SM-Soil moisture, SPEI- Standardised precipitation-evapotranspiration index, SP-Solar radiation, T-Temperature) for plot categories (SS-South slope, NS-North slope, PL-Plateau, VA-Valley) for previous and current year (transparent and solid color, respectively). Dashed lines indicate statistical significance (p<0.05).


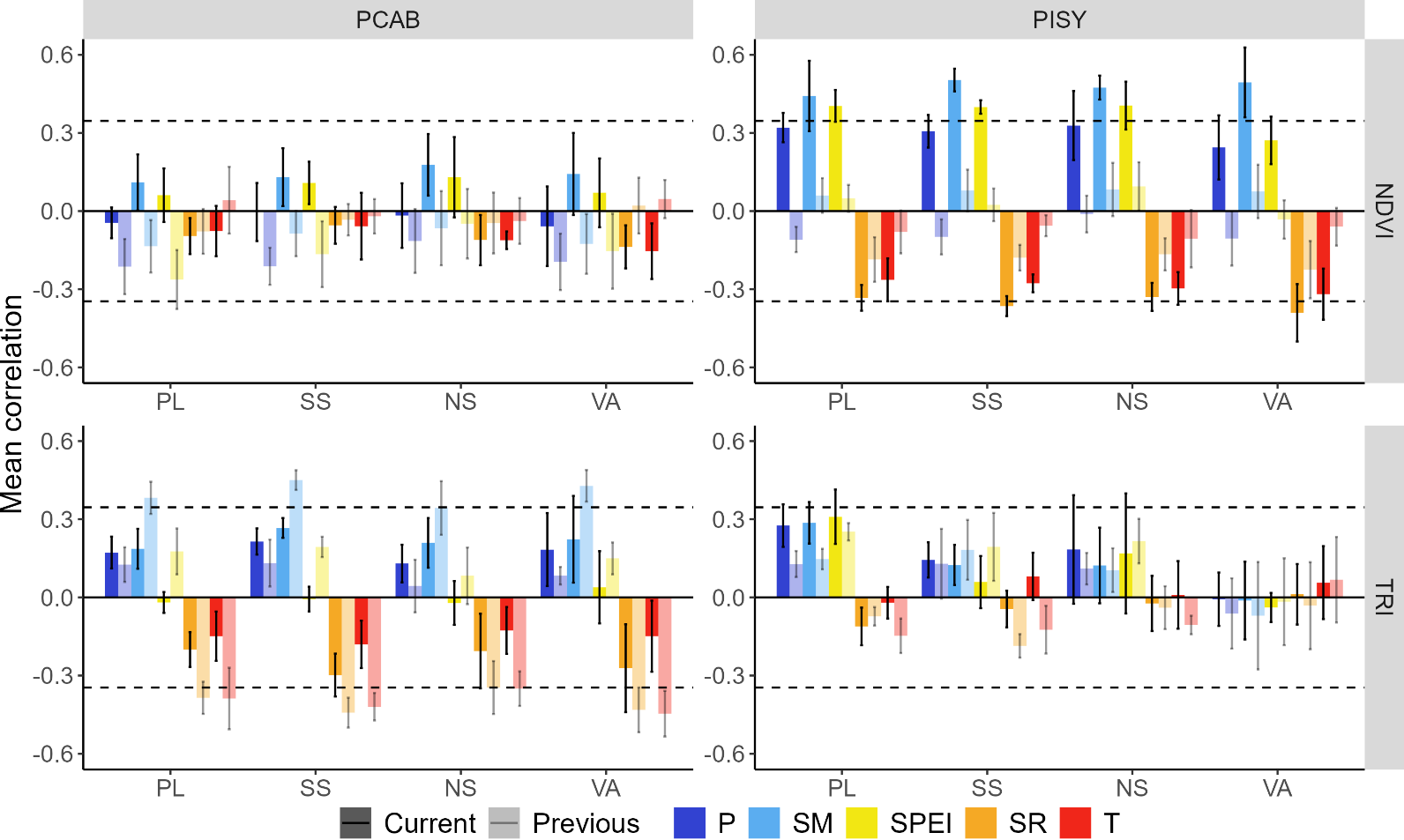


**Text S1:** To quantify the resiliency of the biomass compartments in response to dry spells, we calculated three resilience indices following Lloret et al. (2011): resilience (RS = post-drought growth/pre-drought growth), resistance (RT = growth in drought year/pre-drought growth), and recovery (RC = growth in drought year/post-drought growth). We considered four years pre- and post-drought, and we used for the calculations the R package pointRes 2.0.1 (van der Maaten-Theunissen et al. 2015). The RS indicates the capacity to achieve the pre-drought level of growth, the RT represents the ability to retain normal growth rates during drought and RC denotes post-drought growth relative to the growth depression during the drought event (Lloret et al. 2011). We applied the Wilcox pairwise test to determine differences in resilience components between drought years (Fig. S5, Table S2).

**Table S3:** Differences in resilience indices: resilience (RS), resistance (RT), and recovery (RC) of normalized difference vegetation index (NDVI) and tree-ring width (TRI) among individual drought years for Pinus sylvestris (PISY) and Picea abies (PCAB) and their plot categories (SS-South slope, NS-North slope, PL-Plateau, VA-Valley) indicated by the Wilcox pairwise post hoc test. Red font denotes statistical significance (p<0.05).

|  |  |  | **RS** | | | | **RT** | | | | **RC** | | | |
| --- | --- | --- | --- | --- | --- | --- | --- | --- | --- | --- | --- | --- | --- | --- |
| **SPECIES** | **VAR** | **YEAR** | **NS** | **PL** | **SS** | **VA** | **NS** | **PL** | **SS** | **VA** | **NS** | **PL** | **SS** | **VA** |
| **PISY** | **TRI** | **1994-2003** | 0.024 | 0.024 | 0.024 | 0.048 | 0.222 | 0.032 | 0.024 | 0.095 | 0.095 | 0.024 | 0.024 | 0.032 |
|  |  | **1994-2006** | 0.841 | 0.032 | 0.310 | 0.548 | 0.048 | 0.024 | 0.032 | 0.024 | 0.024 | 0.151 | 0.024 | 0.024 |
|  |  | **2003-2006** | 0.024 | 0.024 | 0.111 | 0.048 | 0.111 | 0.095 | 0.151 | 0.024 | 0.024 | 0.024 | 0.024 | 0.024 |
|  | **NDVI** | **1994-2003** | 0.024 | 0.024 | 0.024 | 0.024 | 0.167 | 0.286 | 1.000 | 1.000 | 1.000 | 0.024 | 0.024 | 0.286 |
|  |  | **1994-2006** | 0.310 | 0.024 | 0.024 | 0.024 | 0.619 | 0.444 | 1.000 | 1.000 | 1.000 | 0.151 | 0.190 | 0.286 |
|  |  | **2003-2006** | 0.024 | 0.024 | 0.024 | 0.095 | 0.619 | 0.444 | 1.000 | 1.000 | 1.000 | 0.063 | 0.190 | 1.000 |
| **PCAB** | **TRI** | **1994-2003** | 0.421 | 0.095 | 0.310 | 0.095 | 0.024 | 0.024 | 0.024 | 0.024 | 0.024 | 0.024 | 0.024 | 0.048 |
|  |  | **1994-2006** | 0.024 | 0.024 | 0.024 | 0.032 | 0.095 | 0.024 | 0.024 | 0.222 | 0.095 | 0.222 | 0.095 | 0.841 |
|  |  | **2003-2006** | 0.032 | 0.024 | 0.024 | 0.024 | 0.024 | 0.024 | 0.024 | 0.032 | 0.063 | 0.024 | 0.032 | 0.444 |
|  | **NDVI** | **1994-2003** | 0.024 | 0.095 | 0.095 | 0.095 | 0.310 | 0.421 | 0.032 | 0.421 | 0.024 | 0.619 | 0.690 | 0.444 |
|  |  | **1994-2006** | 0.024 | 0.302 | 0.190 | 0.111 | 0.048 | 0.167 | 0.024 | 0.048 | 1.000 | 0.619 | 0.032 | 0.444 |
|  |  | **2003-2006** | 0.421 | 0.548 | 1.000 | 1.000 | 0.063 | 0.302 | 0.032 | 0.111 | 0.111 | 0.452 | 0.024 | 0.286 |

**Figure S5:** Boxplots of resilience indices: resilience (RS), resistance (RT), and recovery (RC) of normalized difference vegetation index (NDVI; upper panel) and tree-ring width (TRI; lower panel) in individual dry years between plot categories (color; SS-South slope, NS-north slope, PL-Plateau, VA-Valley) for Pinus sylvestris (PISY; right panel) and Picea abies (PCAB; left panel).


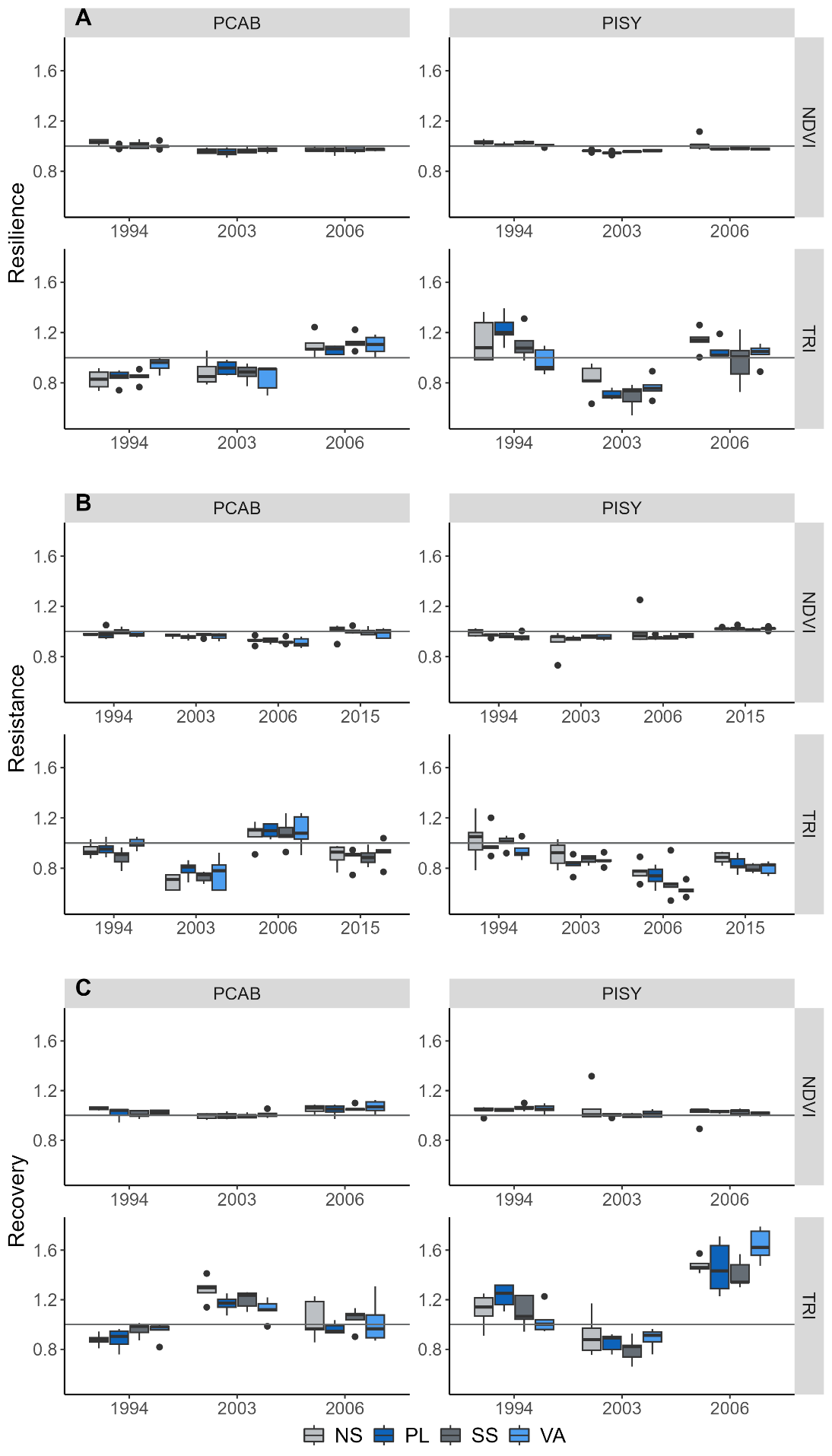

Supplement: Supplementary file 1 — Supplementary file1 (DOCX 1665 KB) [file 484_2024_2682_MOESM1_ESM.docx]
